# Evidenzbasierte Public Health: Perspektiven und spezifische Umsetzungsfaktoren

**DOI:** 10.1007/s00103-021-03308-x

**Published:** 2021-04-13

**Authors:** Eva A. Rehfuess, Ana Zhelyazkova, Peter von Philipsborn, Ursula Griebler, Freia De Bock

**Affiliations:** 1 Lehrstuhl für Public Health und Versorgungsforschung, Institut für medizinische Informationsverarbeitung, Biometrie und Epidemiologie, Ludwig-Maximilians-Universität, Marchioninistr. 17, 81377 München, Deutschland; 2 Pettenkofer School of Public Health, München, Deutschland; 3 Institut für Notfallmedizin und Medizinmanagement (INM), Klinikum der Universität München, München, Deutschland; 4 Department für Evidenzbasierte Medizin und Evaluation, Donau-Universität Krems, Krems, Österreich; 5 Bundeszentrale für gesundheitliche Aufklärung (BZgA), Köln, Deutschland

**Keywords:** Modell, Prävention, Gesundheitsförderung, Definition, Konzept, Model, Prevention, Health promotion, Definition, Concept

## Abstract

Evidenzbasierung stellt auch im öffentlichen Gesundheitswesen eine zentrale Anforderung dar. 5 allgemeine Prinzipien gelten in der evidenzbasierten Medizin wie in der evidenzbasierten Public Health (EBPH). Sie umfassen Systematik, Transparenz im Umgang mit Unsicherheit, Integration und Partizipation, Umgang mit Interessenkonflikten sowie einen strukturierten, reflektierten Prozess. Dieser Artikel hat das Ziel, ein gemeinsames Verständnis von evidenzbasierter Public Health zu fördern.

Mit einer systematischen Literaturrecherche zu Perspektiven der Evidenzbasierung in Public Health wurde das internationale Verständnis von EBPH beleuchtet. So wurden 20 englischsprachige und eine deutschsprachige Quelle identifiziert und einer Inhaltsanalyse unterzogen. Über die allgemeinen Prinzipien hinausgehende Charakteristika dieser Perspektiven befassen sich mit Aspekten der Bevölkerungsorientierung, dem zugrunde liegenden Verständnis von Evidenz, zu beteiligenden Disziplinen und Interessengruppen sowie relevanten Kriterien und notwendigen Kompetenzen für eine Umsetzung von Evidenzbasierung.

Darauf aufbauend und unter Berücksichtigung methodischer Entwicklungen im Umgang mit Komplexität lassen sich 4 spezifische Umsetzungsfaktoren der EBPH ableiten. Sie umfassen Theorie, Interdisziplinarität, Kontextabhängigkeit und Komplexität sowie allgemeine gesellschaftliche Aspekte.

Die praktische Umsetzung von EBPH erfordert personelle und finanzielle Ressourcen sowie Kompetenzen – unter anderem für die Durchführung systematischer Übersichtsarbeiten zur Wirksamkeit von Maßnahmen unter Alltagsbedingungen, für die wissenschaftliche Betrachtung weiterer relevanter Fragestellungen sowie für die Etablierung transparenter Prozesse zur Formulierung von Empfehlungen.

**Zusatzmaterial online:**

Zusätzliche Informationen sind in der Online-Version dieses Artikels (10.1007/s00103-021-03308-x) enthalten.

## Abstract (en)

An evidence-based approach is considered a central requirement in the public health system. Five general principles apply in evidence-based medicine as well as in evidence-based public health (EBPH). These comprise a systematic approach, transparency in relation to uncertainty, integration and participation, management of conflicts of interest, and a structured, reflective process. This article aims to promote a common understanding of what taking an evidence-based approach entails in the field of public health.

We explored the international understanding of EBPH through systematic literature searches of definitions, concepts, and models regarding an evidence-based approach in public health. In this manner, 20 English sources and one German source were identified and examined using content analysis. Beyond the above-described general principles, characteristics of these perspectives are concerned with a population orientation, the underlying concept of evidence, disciplines, and stakeholder groups to be involved in the process, as well as relevant criteria and necessary competencies for implementing an evidence-based approach.

Based on this, and taking into account methodological developments to address complexity, four specific implementation factors of EBPH can be derived. These include theory, interdisciplinarity, context-sensitivity, and complexity as well as general societal aspects.

The practical implementation of EBPH requires human and financial resources as well as competencies – among others, for conducting systematic reviews of the effectiveness of measures, examining other relevant questions in a scientific manner, and establishing transparent processes to formulate recommendations.

## Einleitung

Der Begriff der Evidenzbasierung hat seine Ursprünge in der Medizin, wo das Konzept bereits in den 1990er-Jahren geprägt wurde und sich seitdem als internationale Bewegung etabliert hat. Evidenzbasierte Medizin (EBM) beschreibt die Integration von bestverfügbaren wissenschaftlichen Erkenntnissen, klinischer Expertise, Werten und Präferenzen von PatientInnen [1]. Das Herzstück der EBM ist die Betonung einer verlässlichen wissenschaftlichen Basis von klinischen Entscheidungen. Inhaltlich gab es aber während der letzten 30 Jahre vielfältige Weiterentwicklungen, vor allem wird zunehmend die Bedeutung der Werte und Präferenzen von PatientInnen in Richtung partizipative Entscheidungsfindung („shared decision-making“) betont und umgesetzt [2, 3].

Evidenzbasierung stellt auch in der öffentlichen Gesundheit oder Public Health mit ihren vielfältigen Aktivitäten hinsichtlich Prävention, Gesundheitsförderung und Gesundheitsschutz eine zentrale Anforderung dar. Insbesondere muss die Wirksamkeit von Public-Health-Maßnahmen auf Bevölkerungsebene und unter Alltagsbedingungen sorgfältig beleuchtet werden. Außerdem sollten potenziell negative Auswirkungen möglichst gering ausfallen, denn auch Public-Health-Maßnahmen können eine Vielzahl von nichtintendierten Folgen, die positiv oder negativ ausfallen können, haben. Analog zu EBM sind also Wirksamkeit *und* Sicherheit von Bedeutung, der Nachweis eines Nettonutzens (d. h. Nutzen > Schaden) einer Maßnahme ist somit eine wichtige Voraussetzung für eine evidenzbasierte Public Health (EBPH). Darüber hinaus betreffen Public-Health-Maßnahmen große Bevölkerungsgruppen und diverse Lebensbereiche und können unter Umständen individuelle Freiheiten beschneiden, weshalb die Schaden-Nutzen-Abwägung noch umfassender verstanden werden muss als in der EBM [4].

Die Bedeutung von Qualitätssicherung und damit auch von Evidenzbasierung hinsichtlich der Kernhandlungsfelder von Public Health ist im deutschen Gesundheitssystem an vielen Stellen verankert. So schreibt zum Beispiel das deutsche Sozialgesetzbuch vor, dass alle bei der gesetzlichen Krankenversicherung erstattungsfähigen Gesundheitsleistungen nach den Prinzipien der EBM bewertet werden müssen, u. a. auf Grundlage der wissenschaftlichen Expertisen des Instituts für Qualität und Wirtschaftlichkeit im Gesundheitswesen (IQWiG; [5]). Auch bei der Umsetzung des im Jahr 2015 verabschiedeten Präventionsgesetzes wird auf Evidenz und Qualitätsorientierung gesetzt, hier sind insbesondere die Bundesrahmenempfehlungen der Nationalen Präventionskonferenz von Bedeutung [6]. Eine einheitliche Grundlage und Operationalisierung, auf die sich alle relevanten Akteure aus Praxis, Politik und Wissenschaft in Deutschland beziehen können, sind deshalb dringend notwendig. Hier setzt ein vor Kurzem veröffentlichtes Memorandum zu evidenzbasierter Prävention und Gesundheitsförderung der Bundeszentrale für gesundheitliche Aufklärung (BZgA; [7]) an mit dem Ziel, einen Standard für das Verständnis und die Umsetzung von Evidenzbasierung in Deutschland zu setzen.

Aus der EBM lassen sich allgemeine Prinzipien von Evidenzbasierung ableiten; diese gelten auch für die EBPH. Zudem lassen sich diese Prinzipien in weiteren Gesundheitsberufen, wie Pflege und Physiotherapie [8], sowie in Sektoren und Wissenschaftsfeldern wie Bildung [9, 10], Psychologie [11], Management [12] oder Politik [13] anwenden. Diese Prinzipien werden je nach Quelle unterschiedlich aufgeführt und kategorisiert [2]. In dem bereits erwähnten Memorandum der BZgA werden diese allgemeinen Prinzipien als 5 STIIP-Prinzipien beschrieben (Tab. 1). Diese beinhalten *Systematik (S), Transparenz und Umgang mit Unsicherheit (T), Integration und Partizipation (I), Umgang mit Interessenkonflikten (I) *und* strukturierter, reflektierter Prozess (P).*

**Table 1:** 

S	*Systematik*	Wesentlich für eine evidenzbasierte Entscheidung sind die systematische Sichtung, Bewertung und Zusammenfassung der besten verfügbaren wissenschaftlichen Erkenntnisse. Eine Entscheidung basierend auf einer einzelnen Studie (wenn mehr als eine relevante Studie vorliegt) oder der selektiven Auswahl von Studienergebnissen widerspricht dem Prinzip der Systematik
		Systematische Übersichtsarbeiten oder eine komprimierte Form dieser Methode wie Rapid-Reviews, Evidence Maps oder Overviews of Systematic Reviews setzen dieses Prinzip in der Praxis um
T	*Transparenz im Umgang mit Unsicherheit*	Jede Entscheidung birgt Unsicherheiten in sich – die transparente Darstellung des zugrunde liegenden Vorgehens legt diese Unsicherheiten offen. Dies ermöglicht eine kritische Prüfung des Entscheidungsfindungsprozesses an sich sowie der Glaubwürdigkeit der verwendeten Evidenz
		Das Prinzip von Transparenz im Umgang mit Unsicherheit kann auf mehreren Ebenen umgesetzt werden: – Durch einen explizit gestalteten Prozess für die Entscheidungsfindung, wie zum Beispiel bei Leitlinien – Durch eine vorab festgelegte und klar beschriebene Methodik für die Zusammenführung von wissenschaftlichen Erkenntnissen und der Bewertung der Qualität verwendeter Studien, insbesondere im Rahmen von systematischen Übersichtsarbeiten und – Durch die systematische und transparente Bewertung von Unsicherheit in der verwendeten Evidenz, wie zum Beispiel durch Evidenzgrade dargestellt
I	*Integration und Partizipation*	Evidenzbasierte Entscheidungen sind nicht allein von Wissenschaftlichkeit geprägt, sondern sollen die Kompetenz und Erfahrung von Verantwortlichen sowie die Werte und Präferenzen von Betroffenen einbeziehen. In der evidenzbasierten Medizin findet diese Integration in Form einer partizipativen Entscheidungsfindung („shared decision-making“) von BehandlerIn und PatientIn statt. Evidenzbasierte Public Health ist durch eine Vielfalt von Interessengruppen in unterschiedlichen gesellschaftlichen Sektoren gekennzeichnet – darunter diejenigen, die eine Maßnahme finanzieren oder umsetzen, und diejenigen, die direkt oder indirekt von dieser Maßnahme betroffen sind
		Die Umsetzung des Prinzips der Integration und Partizipation erfolgt darüber, wer in den Entscheidungsprozess involviert ist und in welcher Form die Beteiligung erfolgt – ob durch eine repräsentative Umfrage, Konsultationen oder eine konkrete Mitwirkung an der Entscheidung
I	*Umgang mit Interessenkonflikten*	Bei Entscheidungen zu Public-Health-Maßnahmen müssen diverse, für sich genommen legitime Interessen in Einklang gebracht werden. Deshalb sollte die Rolle verschiedener Interessen in Entscheidungsprozessen transparent gemacht werden
		Interessen – darunter finanzielle, institutionelle, verwandtschaftliche und viele andere – müssen nach den wissenschaftlichen Regeln des Umgangs mit Interessenkonflikten offengelegt werden. Interessenkonflikte, die das Risiko für eine systematisch verzerrte Beurteilung der Evidenz (Bias) nennenswert erhöhen, sollten minimiert werden. Bei schwerwiegenden Interessenkonflikten sollte eine Person vom Entscheidungsprozess ausgeschlossen werden
P	*Strukturierter, reflektierter Prozess*	Evidenzbasierung ist durch einen strukturierten Prozess gekennzeichnet. Dieser Prozess besteht aus 5 Schritten: (1) Formulierung einer klaren Fragestellung; (2) Suche nach der besten verfügbaren Evidenz; (3) kritische Prüfung der wissenschaftlichen Erkenntnisse hinsichtlich Glaubwürdigkeit und Relevanz; (4) Anwendung der Evidenz – ob am Krankenbett, bei der Entwicklung einer gesundheitsförderlichen Maßnahme oder im Rahmen einer gesundheitspolitischen Entscheidung; (5) Bewertung der Umsetzung – ob durch kritische Reflexion oder begleitende Evaluation

Die Operationalisierung dieser Prinzipien ist in Tab. 1 kurz dargestellt. Eine ausführlichere Beschreibung der methodischen Vorgehensweisen für die Erhebung und Bewertung von Evidenz sowie der etablierten Verfahren zur Entscheidungsfindung findet sich im Memorandum der BZgA [7].

Dieser Artikel hat das Ziel, ein gemeinsames Verständnis von evidenzbasierter Public Health zu fördern – basierend auf dem Memorandum der BZgA, das auf Prävention und Gesundheitsförderung fokussiert, und weiteren internationalen Quellen, die sich mit allen Bereichen von Public Health befassen. Der Artikel sichtet systematisch internationale Definitionen, Konzepte und Modelle von Evidenzbasierung in Public Health und diskutiert auf dieser Basis die Notwendigkeit einer Weiterentwicklung der Herangehensweisen der EBM für eine EBPH durch die Entwicklung von public-health-spezifischen Umsetzungsfaktoren.

## Verständnis von Evidenzbasierung in Public Health

Die in der Einleitung beschriebenen STIIP-Prinzipien bilden eine wichtige Grundlage für eine evidenzbasierte Public Health. Allerdings stellen sich in Hinblick auf ihre Anwendung und Umsetzung eine Reihe von Fragen: Inwieweit decken sich diese allgemeinen Prinzipien mit dem Verständnis von EBPH in der Literatur? Gibt es – für eine EBPH – Aspekte, die durch die oben genannten Prinzipien noch nicht ausreichend abgedeckt sind? Um sich diesen Fragen anzunähern, wurde eine systematische Literaturrecherche zu Perspektiven der Evidenzbasierung in Public Health durchgeführt. Diese hatte das Ziel, Aufschluss über die Verwendung unterschiedlicher Begrifflichkeiten und die ihnen zugrunde liegenden Definitionen, Konzepte und Modelle zu geben.

### Methoden

#### Literatursuche

Die Literatursuche nach Büchern, Artikeln in Fachzeitschriften sowie Berichten von ausgewählten Public-Health-Organisationen wurde im Zeitraum vom 25.04.2019 bis zum 15.05.2019 durchgeführt. Die dreistufige Suchstrategie wurde mit der Unterstützung einer Informationsspezialistin (IK) entwickelt und deckte deutsch- und englischsprachige Literatur in den Datenbanken WorldCat, Google Scholar, Google Books und PubMed Central ab. Sie wurde durch google-basierte Internetrecherchen nach Berichten relevanter Public-Health-Organisationen ergänzt. Literatursuche und Screening wurden von einer Autorin (AZ) unter Rücksprache mit 2 weiteren Autorinnen (UG, ER) durchgeführt. Detaillierte Einschlusskriterien für Titel und Abstracts sowie Volltexte wurden vorab festgelegt.

Im ersten Schritt wurden die elektronische Büchersammlung Google Books und der weltweite Verbundkatalog WorldCat anhand einer Kombination von Suchbegriffen zu „Evidenz“ und zu „Public Health“, „Prävention“ und „Gesundheitsförderung“ durchsucht. In WorldCat wurden angesichts der großen Anzahl der so identifizierten Quellen nur die ersten 200 Treffer gescreent. Alle Bücher, die „public health“, „health promotion“, „Gesundheitsförderung“, „prevention“ oder „Prävention“ im Titel führen, wurden einem Screening ihres Inhaltsverzeichnisses oder Abstracts unterzogen. Bücher, die laut Inhaltsverzeichnis oder Abstract einen Schwerpunkt auf Evidenzbasierung setzen, wurden anschließend einem Volltextscreening unterzogen. Eingeschlossen wurden alle Bücher, deren Volltexte und/oder Glossare Definitionen, Modelle, Konzepte oder Perspektiven von Evidenzbasierung im Kontext von Public Health, Gesundheitsförderung oder Prävention beschreiben.

Im zweiten Schritt wurde eine Schneeballsuche (Forward Citation Tracking) in Google Scholar und PubMed Central durchgeführt, basierend auf einem Startset von 5 wissenschaftlichen Fachartikeln [14–18]. Diese waren in den im ersten Schritt eingeschlossenen Büchern als relevante Quellen für EBPH-Perspektiven aufgeführt worden. Das Screeningverfahren wurde anhand der bereits beschriebenen Einschlusskriterien für Titel, Abstracts und Volltexte durchgeführt.

In einem dritten Schritt wurden online zugängliche Quellen der folgenden Public-Health-Organisationen durchsucht: European Centre for Disease Prevention and Control (Europa), Public Health England und National Institute for Health and Care Excellence (Vereinigtes Königreich), Canadian Health Services Research Foundation und National Collaborating Centre for Methods and Tools Canada (Kanada), US Department of Health and Human Services, Centre for Disease Control and Prevention, American Public Health Association (Vereinigte Staaten) und die Weltgesundheitsorganisation (WHO). Angelsächsische Länder lagen dabei im Fokus, da dort eine Evidenzbasierung in der öffentlichen Gesundheit deutlich ausgeprägter und früher erfolgt ist als in anderen Ländern. Die Suche erfolgte über Google anhand der manuellen Eingabe der Suchbegriffe „evidence“, „evidence-based“, „evidence-informed“ kombiniert mit „public health“, „health promotion“, „prevention“, ergänzt durch den Namen bzw. die Abkürzung der Public-Health-Organisation. Die jeweils ersten 20 Treffer wurden anhand der bereits genannten Einschlusskriterien gesichtet.

#### Datenextraktion und Inhaltsanalyse

Die Datenextraktion durch eine Autorin (AZ) erfolgte in Microsoft Excel (Microsoft Corporation, Redmond, WA, USA). Extrahiert wurden Informationen zur Quelle selbst (AutorInnen, Jahr, Art der Quelle) sowie relevante Textpassagen zu Definitionen, Konzepten, Modellen und Perspektiven von Evidenzbasierung in Public Health. Die induktive Inhaltsanalyse wurde in Anlehnung an Elo und Kyngäs durchgeführt [19]. Nach einer anfänglich offenen Codierung der Textauszüge wurde ein Codiersystem erstellt. Dieses wurde getestet und in Abstimmung mit weiteren Autorinnen (UG, ER) diskutiert und überarbeitet und anschließend zum endgültigen Codieren aller Textauszüge verwendet. Die Inhaltsanalyse wurde von einer Autorin (AZ) durchgeführt; Unklarheiten wurden mit 2 weiteren Autorinnen (UG, ER) diskutiert, ebenso wurde die Interpretation der Ergebnisse gemeinsam vorgenommen.

### Ergebnisse

**Figure 1:**
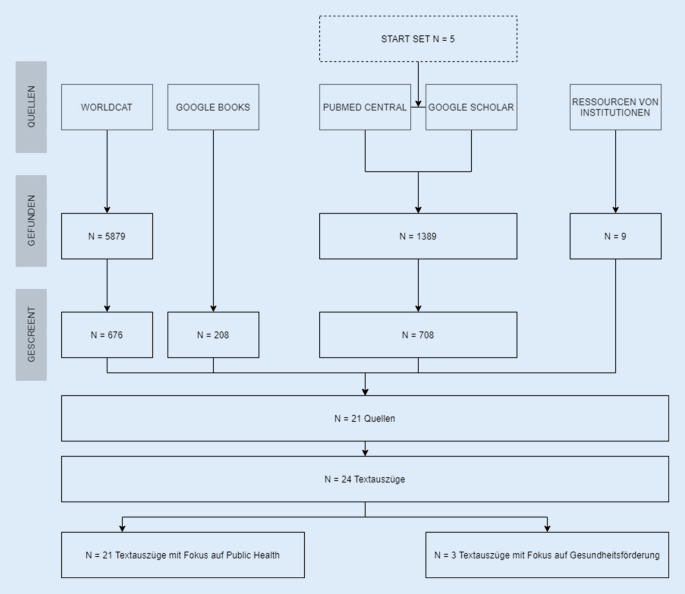

Die Literatursuche führte zu insgesamt 7485 Treffern (WorldCat: 5879; Google Books: 208; PubMed Central/Google Scholar: 1389; Ressourcen von Institutionen: 9). Von diesen entsprachen 21 Quellen den Einschlusskriterien (Abb. 1). 5 dieser Quellen wurden im ersten Schritt identifiziert, weitere 12 Quellen im zweiten Schritt und 4 Quellen im dritten Schritt.

Die Quellen wurden zwischen 1997 und 2018 auf Englisch (20 Quellen) und Deutsch (1 Quelle) publiziert und stammten aus 7 Ländern (8 aus Großbritannien, 7 aus USA, 2 aus Kanada und jeweils eine Quelle aus Deutschland, Schweden, Italien und Japan). Sie enthielten 24 relevante Textauszüge; bei 3 Quellen [20–22] wurden jeweils 2 unterschiedliche Textauszüge extrahiert und separat codiert. 21 Textauszüge aus 18 Quellen befassen sich allgemein mit Evidenzbasierung in Public Health [14, 15, 17, 18, 20–33] und 3 spezifisch mit Evidenzbasierung in der Gesundheitsförderung [34–36]; Quellen, die sich spezifisch mit Evidenzbasierung in der Prävention beschäftigen, wurden nicht gefunden.

Die eingeschlossenen Quellen beinhalten sowohl knapp formulierte Definitionen als auch umfassender beschriebene Konzepte und Modelle von Evidenzbasierung in Public Health und Gesundheitsförderung. Relevante Textauszüge der Perspektiven von Evidenzbasierung in Public Health werden im Onlinematerial in Tab. Z1 aufgeführt. Im Folgenden werden diese zusammenfassend als „Perspektiven von Evidenzbasierung“ bezeichnet. Ausgewählte Charakteristika dieser Textauszüge sind im Onlinematerial in Tab. Z2 dargestellt und/oder werden in den nächsten Abschnitten anhand der folgenden Kategorien des Codiersystems näher beschrieben: Bezeichnung und Bevölkerungsorientierung, Evidenzbasierung als Prozess, beteiligte Disziplinen, Verständnis von Evidenz und relevante Kriterien, Kompetenzen für Evidenzbasierung und Einbindung von Interessengruppen.

#### Bezeichnung und Bevölkerungsorientierung

Am häufigsten wird die Bezeichnung „evidence-based public health“ verwendet [14, 15, 17, 18, 24, 25, 27–29, 32]. Weitere Begriffe sind „evidence-based decision-making“ [23, 24, 33], „evidence-based practice“ [26], „evidence-based policy and practice“ [20, 31], „evidence-informed policy and practice“ [20], „evidence-based approach in public health practice“ [21], „evidence-informed public health“ [30], „evidence-informed decision-making in public health“ [22, 30], „knowledge-based public health“ [21] und – für den Bereich Gesundheitsförderung – „evidence-based health promotion“ [34–36]. Insgesamt lassen sich keine wesentlichen Unterschiede zwischen den auf Gesundheitsförderung eingeschränkten und den breiteren Public-Health-Perspektiven feststellen.

Erwähnenswert sind konzeptionelle Unterschiede zwischen den Begriffen „evidence-based“ und „evidence-informed“: Der Begriff „evidence-based“ – wie zum Beispiel bei Orme [20] oder Brownson et al. [22] verwendet – geht meist mit einem eher engen, epidemiologischen Verständnis von Evidenz sowie einer relativ starken Gewichtung dieser Evidenz in Entscheidungsprozessen einher. Der Begriff „evidence-informed“ spiegelt hingegen meist ein interdisziplinäres und methodisch offenes Verständnis von Evidenz sowie eine weniger starke Gewichtung dieser Evidenz in Entscheidungsprozessen wider; in evidenz-informierten Prozessen spielen neben wissenschaftlichen Erkenntnissen explizit politische, wirtschaftliche, organisatorische und andere Faktoren eine wesentliche Rolle [20, 22].

2 Textauszüge beschreiben die Ursprünge dieses Prozesses in der evidenzbasierten Medizin [14, 17] oder beziehen sich wörtlich auf deren Definition von David Sackett [1], so auch die für den europäischen Kontext besonders relevante Definition des European Centre for Disease Prevention and Control:

Evidence-based public health could be defined as integrating the best available evidence with the knowledge and considered judgements from stakeholders and experts to improve health and protect the population from infectious and environmental hazards [29].

Die meisten Perspektiven benennen die Orientierung an der Bevölkerung im Gegensatz zu einer Orientierung am Individuum als wesentlichen Unterschied zwischen EBM und EBPH. In mehreren Quellen wird präzisierend zu einem Bevölkerungsfokus die Abhängigkeit der Wirksamkeit oder Umsetzbarkeit von Maßnahmen von unterschiedlichen geografischen oder soziokulturellen Kontexten beschrieben [20, 22, 26, 30, 33]. Eine Quelle weist außerdem darauf hin, dass Public-Health-Maßnahmen oft auf mehreren Ebenen durchgeführt und beeinflusst werden können und so zum Beispiel im Zusammenspiel von zwischenmenschlichen Dynamiken mit dem Setting wirken [35].

#### Evidenzbasierung als Prozess

Evidenzbasierung wird in 11 von 24 Textauszügen als ein Prozess beschrieben; weitere Textauszüge verwenden Bezeichnungen wie „endeavour“ [17] oder „situation“ [20]. 4 Textauszüge beschreiben konkrete Phasen wie Problemidentifikation, Projektentwicklung, -implementierung und -evaluation [15, 24, 25, 34], so auch die 1997 formulierte und damit wohl älteste Definition des Begriffs „evidence-based public health“ von Jenicek:

As in evidence-based medicine, its steps should be: formulation of a clear question from a public health problem; searching for evidence; appraisal of evidence; selection of the best evidence for a public health decision; linking evidence with public health experience, knowledge, and practice; implementation of useful evidences in public health practice (policies and programs); evaluation of such implementations and of the overall performance of the evidence-based public health practitioner, and teaching others how to practice evidence-based public health [14].

5 Textauszüge betonen die Bedeutung der Dissemination und Kommunikation relevanter Erkenntnisse als finale Phase des Prozesses [22, 27, 30, 36]. In der Definition des National Collaborating Centre for Methods and Tools in Kanada heißt es:

The process of distilling and disseminating the best available evidence from research, context and experience … [30].

#### Beteiligte Disziplinen

Insgesamt 14 Textauszüge beinhalten Hinweise auf relevante Disziplinen. Ausgehend von der Epidemiologie werden am häufigsten Bezüge zu den Wirtschaftswissenschaften hergestellt [15, 21, 28, 35] ebenso wie zur Versorgungsforschung [15, 23] und zu Verhaltens- und Sozialwissenschaften [15, 17, 20, 24, 25, 31, 35]. Ein Beispiel hierfür ist die Definition von Brownson et al. [15], wobei Brownson als einer der wichtigsten Vertreter von EBPH in den USA gilt:

The authors define EBPH as the development, implementation, and evaluation of effective programs and policies in public health through application of principles of scientific reasoning including systematic uses of data and information systems and appropriate use of program planning models. In EBPH, the most viable approach to a public health problem is chosen from among a set of rational alternatives. This process relies on several related disciplines including epidemiology, biostatistics, behavioral sciences, health economics, and health care management.

Satterfield et al. vereinen Methoden und Praktiken aus unterschiedlichen Fachbereichen (Pflegewissenschaften, Public Health, Sozialarbeit, Psychologie) in einem transdisziplinären Modell [26]:

Our … model … has a transdisciplinary perspective. It incorporates each discipline’s most important advances and attempts to address remaining deficiencies. The model is grounded in an ecological framework and emphasizes shared decision making. … Both the impact on the population and health maintenance can be enhanced by intervening also at the interpersonal, organizational, community, and public policy levels.

#### Verständnis von Evidenz und relevante Kriterien

Die eingeschlossenen Textauszüge verwenden verschiedene Konzepte von Evidenz, die sich unter anderem darin unterscheiden, wie breit bzw. eng der Begriff der Evidenz ausgelegt wird. An einem Ende dieses Spektrums steht ein Verständnis von Evidenz als wissenschaftlicher Wirksamkeitsbeleg [20, 23, 24, 26, 31], zum Beispiel formuliert als „scientific evidence that demonstrates effectiveness“ [24].

Bei einem zunehmend breiten Verständnis von Evidenz kommen wissenschaftliche Erkenntnisse zu weiteren Fragestellungen hinzu [14, 15, 17, 18, 20–22, 25, 27–30, 32, 33, 35, 36], so auch in der deutschsprachigen Definition von Gerhardus et al.:

Evidence-based Public Health soll die Gesundheit auf Bevölkerungsebene durch wissenschaftlich abgesicherte Entscheidungen verbessern. Dafür wird das verfügbare Wissen der medizinischen, ökonomischen, ethischen, soziokulturellen und rechtlichen Aspekte von Krankheit und Maßnahmen systematisch, transparent und zielgerecht bewertet und in die Entscheidungsprozesse eingebracht. Alle Schritte – von der Problemstellung bis zur Umsetzung der Maßnahmen und Programme – sollen explizit, transparent und begründet sein [28].

Diverse Quellen nennen auch konkrete Kriterien, anhand welcher – neben dem Kriterium der Wirksamkeit – Entscheidungen über Public-Health-Maßnahmen getroffen werden sollen, darunter Effizienz, Angemessenheit, Machbarkeit, Akzeptanz und Kosten-Nutzen-Verhältnis [15, 21, 34, 35].

Am anderen Ende des Spektrums werden auch nichtwissenschaftlich generierte Erkenntnisse („colloquial evidence“ oder „knowledge“) als Evidenz bezeichnet, unter anderem als „experience“ [30], darunter Erfahrungen im politischen oder organisatorischen Bereich [19] oder von Zielpopulationen und der Gesellschaft im Allgemeinen [21]. Smith et al. halten fest: „formal evidence alone is not a sufficient basis for effective health promotion“ [35].

Neben dem Verständnis von Evidenz werden auch konkrete Merkmale von Evidenz benannt. In 11 Textauszügen wird Evidenz von höchster oder bestvorhandener Qualität gefordert [14, 22, 25–27, 29, 30, 32, 33, 36], ein Textauszug verwendet den Begriff „quality research evidence“ [34]. 2 weitere Quellen betonen außerdem die Notwendigkeit aktueller Evidenz [14, 25].

#### Kompetenzen für Evidenzbasierung

7 Quellen stellen notwendige Fähigkeiten und Kompetenzen für eine Evidenzbasierung in der Praxis dar. Neben allgemeinen Public-Health-Kompetenzen [15, 34] wie „data collection and analysis“ [21] und „public health expertise“ [30, 34] werden interdisziplinäre und partizipative Ansätze wie „to record and use the experience of communities and societies“ betont [21]. In einigen Textauszügen werden diese wissenschaftsnahen Kompetenzen durch fachübergreifende Kompetenzen ergänzt, so betonen Magnus et al. die Relevanz von „leadership“ und „community assessment“ [36] und Brownson et al. „enhanced communication, common sense, and political acumen“ [27].

#### Einbindung von Interessengruppen

3 Quellen beschreiben „public health practitioners“ [14, 15] oder „health promotion practitioners“ [34] als die Akteure, die primär verantwortlich für die Umsetzung von Evidenzbasierung sind, und gehen nicht weiter auf andere Interessengruppen ein. 5 Textauszüge betonen, dass die Perspektiven der Zielpopulation aktiv in Entscheidungsprozesse einbezogen werden sollten [18, 21, 25, 30]. Eine weitere Quelle weitet dies auf die Perspektiven anderer relevanter EntscheidungsträgerInnen aus [29]. Gemeinsame Entscheidungsfindung – im Sinne von „shared decision-making“ in der EBM [3] – wird in 5 Textauszügen genannt [22, 26, 27, 32, 36]. Magnus et al. beschreiben außerdem, dass die Zielpopulation proaktiv handeln sollte, d. h., „local communities should actively adopt evidence-based strategies“ [36].

## Ableitung public-health-spezifischer Umsetzungsfaktoren von Evidenzbasierung

4 der oben erläuterten 5 allgemeinen Prinzipien der Evidenzbasierung – Systematik, Transparenz und Umgang mit Unsicherheit, Integration und Partizipation und strukturierter, reflektierter Prozess – sind auch in der Mehrzahl der Perspektiven der Evidenzbasierung in Public Health klar erkennbar (Tab. Z1 im Onlinematerial). Erwähnenswert ist, dass das fünfte Prinzip – Umgang mit Interessenkonflikten – nicht explizit beschrieben wird. Auffällig ist außerdem, dass keine der Perspektiven sich direkt auf Ansätze in Lebenswelten (Settings) bezieht oder konkret ein Einbeziehen von Sektoren außerhalb des Gesundheitssektors im Sinne eines Health-in-all-policies-Ansatzes benennt, obwohl dies Kernaspekte von moderner Public Health und Gesundheitsförderung sind.

Neben den allgemeinen Prinzipien der Evidenzbasierung sind für Public Health noch weitere spezifische Umsetzungsfaktoren von besonderer Bedeutung. Sie umfassen *Theorie (T), Interdisziplinarität (I), Kontextabhängigkeit und Komplexität (KK) *und* allgemeine gesellschaftliche Aspekte (A)* und sind für evidenzbasierte Public-Health-Entscheidungen relevant. Dabei lassen sich die Faktoren *Interdisziplinarität* sowie *allgemeine gesellschaftliche Aspekte* direkt aus den Ergebnissen der systematischen Bestandsaufnahme zu Perspektiven der Evidenzbasierung in Public Health ableiten. *Theorie* und *Kontextabhängigkeit und Komplexität* gehen dagegen auf methodische Arbeiten zum Thema Komplexität in Public Health (insbesondere [37–45]) zurück. Tab. 2 stellt diese Umsetzungsfaktoren unter dem Akronym TIKKA dar und beschreibt ihre Relevanz für die EBPH.

**Table 2:** 

T	*Theorie*	Das Kriterium *Theorie* ist den Wirkmechanismen von Maßnahmen gewidmet. Sie beschreiben, wie Public-Health-Maßnahmen Gesundheit beeinflussen und nichtintendierte und gegebenenfalls schädliche Wirkungen erzeugen können. Diese Wirkungen materialisieren sich oft über viele Zwischenschritte – zum Beispiel durch verringerte Exposition gegenüber einem Umweltrisikofaktor oder durch verstärkt gesundheitsförderndes Verhalten – und in Wechselwirkung mit dem Kontext. Eine fehlende Auseinandersetzung mit den komplexen Wirkmechanismen von Public-Health-Maßnahmen kann ein Grund für mangelnde Wirksamkeit unter Alltagsbedingungen sein. Deshalb sollen sowohl die Entwicklung als auch die Evaluation von Public-Health-Maßnahmen relevante Theorien und Modelle berücksichtigen und den vermuteten Wirkmechanismus beschreiben. Letzteres ist auch dann möglich, wenn keine passenden, spezifischen Theorien vorliegen
		Je nach Maßnahme können zum Beispiel Ursachentheorien aus der Soziologie oder Verhaltensmodelle und Veränderungstheorien aus der Psychologie genutzt werden
I	*Interdisziplinarität*	Das Gelingen von Public-Health-Maßnahmen ist oft auf das Zusammenwirken diverser Disziplinen angewiesen, darunter zusätzlich zu den Gesundheitswissenschaften natur-, sozial- und technikwissenschaftliche Fächer, z. B. Psychologie, Soziologie und Ökonomie. Im Sinne der Methodenpluralität können diverse methodische Vorgehensweisen zum Einsatz kommen und disziplinenübergreifend wissenschaftliche Erkenntnisse zusammengeführt werden
		In Abhängigkeit von Gesundheitsproblem bzw. Art der Maßnahme sollte geprüft werden, welche Disziplinen und gegebenenfalls Sektoren eingebunden werden müssen. Die konkrete Fragestellung bestimmt die jeweils bestmögliche Methodik, wie in den relevanten Disziplinen verankert. Zum Beispiel sind dies bei Fragen zur Akzeptanz einer Maßnahme meist qualitative Methoden, bei Fragen zu den ethischen Implikationen einer Maßnahme ethische Analysen
K	*Kontextabhängigkeit und Komplexität*	Public-Health-Maßnahmen sind oft durch das Zusammenspiel vieler Einzelelemente auf unterschiedlichen Ebenen (zum Beispiel Individuum, Familie, Schule und Stadtviertel) gekennzeichnet und müssen dann als „Interventionen in komplexen Systemen“ begriffen werden. Ihre Wirksamkeit – und auch die Möglichkeiten ihrer Umsetzung – kann je nach den Rahmenbedingungen in Setting und Kontext variieren
		Ein Durchdenken von Komplexität hinsichtlich der Maßnahme und ihrer postulierten Wirkmechanismen und hinsichtlich ihrer möglichen Wechselwirkungen mit dem Kontext ist vor allem in der Planungsphase entscheidend. So kann eine grafische Darstellung in Form eines logischen Modells sicherstellen, dass keine wesentlichen Aspekte vergessen werden. Wo sinnvoll, sollten einzelne Aspekte von Komplexität auch hinsichtlich der Erstellung von Evidenz und ihrer Nutzung in Entscheidungsprozessen weiterverfolgt werden. Ein besonderes Augenmerk auf die unterschiedlichen Dimensionen von Kontext, zum Beispiel anhand existierender Modelle und methodischer Herangehensweisen, hilft dabei, förderliche oder hinderliche Faktoren hinsichtlich der Wirksamkeit und Umsetzung einer Maßnahme zu identifizieren
A	*Allgemeine gesellschaftliche Aspekte*	Public-Health-Entscheidungen betreffen die Bevölkerung im Allgemeinen oder größere Bevölkerungsgruppen. Sie ziehen neben den angestrebten gesundheitlichen Wirkungen oft vielfältige allgemeine gesellschaftliche Folgen nach sich. Neben der Abwägung von gesundheitlichem Nutzen und Schaden spielen deshalb Aspekte wie die Akzeptanz der Maßnahme in der Bevölkerung, die Machbarkeit und Kosten einer Umsetzung, Auswirkungen auf gesundheitliche Chancengleichheit und Umwelt eine große Rolle
		Die Umsetzung dieses Kriteriums erfolgt durch die Integration dieser Aspekte in den Entscheidungsprozess, d. h. konkret durch Aufführung und Betrachtung aller für den Entscheidungsprozess relevanten Aspekte. Idealerweise erfolgt dies durch die Nutzung von festgelegten Entscheidungskriterien im Rahmen eines „evidence to decision framework“

Diese 4 TIKKA-Umsetzungsfaktoren sollten bei der Entwicklung, Pilotierung, Evaluation und Implementierung von Maßnahmen berücksichtigt werden. Sie spielen außerdem eine entscheidende Rolle bei den Prozessen zur Erhebung und Bewertung von Evidenz zur Wirksamkeit sowie zur Erhebung und Bewertung von Evidenz zu weiteren Fragestellungen. Zum Beispiel spielen bei der Bewertung der Wirksamkeit von Public-Health-Maßnahmen auch die zugrunde liegenden Theorien oder Wirkmechanismen (z. B. verhaltenspsychologische oder soziologische Modelle) eine wichtige Rolle, außerdem sollten Kontextabhängigkeiten geprüft werden. Und wenn man solche Maßnahmen in ihrem komplexen Zusammenspiel mit dem System, in dem sie umgesetzt werden, begreift, sollten gesamtgesellschaftliche Aspekte wie nichtintendierte Auswirkungen der Maßnahmen auf andere Sektoren und die Akzeptanz der Maßnahmen bei der Zielgruppe und weiteren Interessengruppen ebenfalls betrachtet werden. Dies erfordert ein interdisziplinäres Methodenpaket. Die Operationalisierung der TIKKA-Umsetzungsfaktoren wird im Memorandum der BZgA ausführlicher erläutert [7].

## Fazit

Für den deutschsprachigen Raum und auch in der internationalen Literatur liegt bisher keine einheitliche, allgemein anerkannte Definition von EBPH vor. In Anlehnung an die EBM-Definition von David Sackett [1] und die EBPH-Definition des European Centre for Disease Prevention and Control [29] könnte eine Definition von EBPH wie folgt lauten:

*Evidenzbasierte Public Health (EbPH) bezeichnet das Fällen von public-health-relevanten Entscheidungen unter Nutzung der jeweils besten verfügbaren wissenschaftlichen Erkenntnisse, der Expertise relevanter Fachleute und Stakeholder und der Werte und Präferenzen der betroffenen Bevölkerung *[46].

Wissenschaftliche Erkenntnisse sind dabei als Informationen zu verstehen, die mithilfe von wissenschaftlich anerkannten Methoden in einem transparenten Prozess generiert wurden. Das schließt Ergebnisse zur Wirksamkeit von Public-Health-Maßnahmen ebenso wie Erkenntnisse zu ihrer Implementierung und organisationalen, ökonomischen oder ethischen Fragen ein. Dabei können unterschiedliche wissenschaftliche Methoden genutzt werden, wie z. B. randomisierte kontrollierte Studien, Beobachtungsstudien, qualitative Studien und Auswertungen von Routinedaten [47].

Bedeutsamer als eine einheitliche Definition von EBPH ist aber ein ihr zugrunde liegendes gemeinsames Verständnis von EBPH. Eine wichtige Grundlage dafür bilden die in der Einleitung dieses Artikels beschriebenen 5 allgemeinen Prinzipien einer Evidenzbasierung oder STIIP-Prinzipien, d. h. Systematik, Transparenz und Umgang mit Unsicherheit, Integration und Partizipation, Umgang mit Interessenkonflikten und strukturierter, reflektierter Prozess. Ergänzend liefern 4 für alle Kernfelder von Public Health entwickelte Umsetzungsfaktoren eine wichtige Orientierung. Diese TIKKA-Umsetzungsfaktoren beschreiben Theorie, Interdisziplinarität, Kontextabhängigkeit und Komplexität sowie allgemeine gesellschaftliche Aspekte. Methoden und Instrumente der Evidenzbasierung können helfen, diese Prinzipien und Umsetzungsfaktoren im Kontext von Public-Health-Maßnahmen in der Praxis umzusetzen. Hierzu zählen systematische Übersichtsarbeiten und von diesen abgeleitete Formate (z. B. Rapid-Reviews und Evidence Maps), evidenz- und konsensbasierte Leitlinien und die 5 Schritte der Evidenzbasierung (Tab. 1). Von Bedeutung sind außerdem Verfahren zur Konsultation von Interessengruppen und strukturierte Vorgehensweisen zur Formulierung von evidenzbasierten Handlungsempfehlungen unter Nutzung von Entscheidungskriterien („evidence to decision framework“).

Ein evidenzbasiertes Entscheiden ist nicht nur für solche Public-Health-Maßnahmen von Bedeutung, die im Rahmen des Sozialversicherungssystems oder mit öffentlicher Förderung umgesetzt werden, sondern auch für Initiativen der Zivilgesellschaft. Die Umsetzung des in diesem Artikel beschriebenen Verständnisses einer EBPH sollte sich an etablierten Verfahren aus dem In- und Ausland orientieren. Wichtige Schritte hierfür werden im Memorandum der BZgA skizziert, allerdings mit Fokus auf Prävention und Gesundheitsförderung [8]. Diese Umsetzung ist nicht ohne Herausforderungen, insbesondere in Hinblick auf den damit verbundenen Mehraufwand an Ressourcen. Konkret erfordert die Durchführung einer systematischen Übersichtsarbeit zur Wirksamkeit erhebliche Ressourcen, allerdings liegen für viele Public-Health-Maßnahmen relevante Übersichtsarbeiten vor und schnellere Formate wie Rapid-Reviews kommen zunehmend zum Einsatz. Auch die systematische wissenschaftliche Betrachtung anderer relevanter Fragestellungen, zum Beispiel zu Akzeptanz, Umsetzbarkeit und nichtintendierten Wirkungen von Maßnahmen, erfordert Zeit sowie methodische Expertise. Formale Leitlinienprozesse zur Formulierung von interdisziplinären, evidenz- und konsensbasierten Public-Health-Empfehlungen, wie sie im angelsächsischen Raum etabliert sind, sind ebenfalls mit Aufwand verbunden. Wünschenswert wäre eine beispielhafte Anwendung anhand von unterschiedlichen Public-Health-Maßnahmen in unterschiedlichen Public-Health-Bereichen, insbesondere auch im Bereich Gesundheitsschutz. Anhand der so gewonnenen Erkenntnisse könnten Prinzipien und Umsetzungsfaktoren nachgebessert sowie Umsetzungshilfen entwickelt werden.

## Supplementary Information


