# Supplementary material for: Evidence-based public health: perspectives and specific implementation factors
Source: Bundesgesundheitsblatt Gesundheitsforschung Gesundheitsschutz. 2021 Apr 13;64(5):514–23. [Article in German] doi: 10.1007/s00103-021-03308-x (PMC8087605; doi:10.1007/s00103-021-03308-x)
Supplement: Supplementary file 1 [file 103_2021_3308_MOESM1_ESM.pdf]

Bundesgesundheitsblatt 5/2021

Elektronisches Zusatzmaterial zum Beitrag:

Evidenzbasierte Public Health: Perspektiven und spezifische Umsetzungsfaktoren

Eva A. Rehfuess[1,2 *], Ana Zhelyazkova[1,2,3 *], Peter von Philipsborn[1,2], Ursula Griebler[4], Freia De Bock[5]

[1] Institut für medizinische Informationsverarbeitung, Biometrie und Epidemiologie, Ludwig-Maximilians-Universität, München

[2] Pettenkofer School of Public Health, München, Deutschland

[3] Institut für Notfallmedizin und Medizinmanagement (INM), Klinikum der Universität München, München, Deutschland

[4] Department für Evidenzbasierte Medizin und Evaluation, Donau-Universität Krems, Österreich

[5] Bundeszentrale für gesundheitliche Aufklärung (BZgA), Köln, Deutschland

* Geteilte Erstautorenschaft

Inhalt:

Tabelle Z1: Perspektiven von Evidenzbasierung in Public Health, Textauszüge nach Erscheinungsdatum

Tabelle Z2: Ausgewählte Charakteristika der Textauszüge

Literaturverzeichnis

Tabelle Z1: Perspektiven von Evidenzbasierung in Public Health, Textauszüge nach Erscheinungsdatum

**AutorInnen (Jahr)**	**Publikation**	**Textauszug**
**Evidenzbasierung in Public Health**		
Jenicek (1997) **[1]**	Epidemiology, Evidence-Based Medicine, and Evidence-Based Public Health *(Artikel)*	*" ...the conscientious, explicit, and judicious use of current best evidence in making decisions about the care of communities and populations in the domain of health protection, disease prevention, health maintenance and improvement (health promotion)". In the same spirit, it is "...the process of systematically finding, appraising, and using contemporaneous research findings as the basis for decisions in public health.*<br>*As in evidence-based medicine, its steps should be:*<br>• *formulation of a clear question from a public health problem;*<br>• *searching for evidence;*<br>• *appraisal of evidence;*<br>• *selection of the best evidence for a public health decision;*<br>• *linking evidence with public health experience, knowledge, and practice;*<br>• *implementation of useful evidences in public health practice (policies and programs);*<br>• *evaluation of such implementations and of the overall performance of the evidence-based public health practitioner, and teaching others how to practice evidence-based public health."*
Brownson, Gurney, Land (1999) **[2]**	Evidence-Based Decision Making in public health *(Artikel)*	*"The authors define EBPH as the development, implementation, and evaluation of effective programs and policies in public health through application of principles of scientific reasoning including systematic uses of data and information systems and appropriate use of program planning models. In EBPH, the most viable approach to a public health problem is chosen from among a set of rational alternatives. This process relies on several related disciplines including epidemiology, biostatistics, behavioral sciences, health economics, and health care management. Any process or method that is established should recognize that public health practitioners often have substantial administrative duties; therefore, EBPH must be time efficient."*

Canadian Health Services Research Foundation<br><br>(2000)<br><br>**[3]**	Health Services Research and evidence-based decision making<br><br>*(Bericht)*	*"In Canada, the term was given more prominence with the imprimatur of the Prime Minister's National Forum on Health in 1997. (…) In the forum's final report it was defined as: 'the systematic application of the best available evidence to the evaluation of options and to decision-making in clinical, management and policy settings.' The foundation has chosen to focus its efforts on the research needs of managers and policy makers. As currently defined, EBDM seems to demand an increase in the availability and use of health services research as 'the evidence' in EBDM."*
Brownson<br><br>(2003)<br><br>**[4]**	Evidence-Based Public Health<br><br>*(Buch)*	*"The definition of evidence-based public health has been broadened to include 'the development, implementation, and evaluation of effective programs and policies in public health through application of principles of scientific reasoning, including systematic uses of data and information systems, and appropriate use of behavioral science theory and program planning models' (Brownson et al., 1999). From such an approach, activities in public health practice are explicitly linked with the underlying scientific evidence that demonstrates effectiveness. Evidence-based public health involves the development and implementation of effective programs and policies."*
Jenicek, Stachenko<br><br>(2003)<br><br>**[5]**	Evidence-based public health, community medicine, preventive care<br><br>*(Artikel)*	*"The process of systematically finding, appraising, and using contemporaneous clinical and community research findings as the basis for decisions in public health or: The conscientious, explicit, and judicious use of current best evidence in making decisions about the care of communities and populations in the domain of health protection, disease prevention, and health maintenance and improvement (health promotion)*<br>*Steps of its practice*<br>*• Formulation of a clear question arising from a public health problem*<br>*• Searching for evidence*<br>*• Appraisal of evidence*<br>*• Selection of the best evidence for a public health decision*<br>*• Linking evidence with public health experience, knowledge, and practice and with the community values and preferences*<br>*• Implementation of useful evidences in public health practice (policies and programs)*<br>*• Evaluation of such implementations and of the overall performance of the EBPH practitioner, and*<br>*• Teaching others how to practice EBPH*<br>*Its goal: The best possible management of health and disease and their determinants at the community level."*

Kohatsu, Robinson, Torner (2004) **[6]**	Evidence-Based Public Health An Evolving Concept *(Artikel)*	*"EBPH is the process of integrating science-based interventions with community preferences to improve the health of populations."*
Rychetnik, Hawe, Waters, Barratt, Frommer (2004) **[7]**	A glossary for evidence based public health *(Artikel)*	*"Evidence based public health can be defined as a public health endeavour in which there is an informed, explicit, and judicious use of evidence that has been derived from any of a variety of science and social science research and evaluation methods (Sackett et al., 1996). The definition highlights two aspects of evidence based public health: (1) the use of a particular type of evidence to inform public health decisions; and (2) an emphasis on clear reasoning in the process of appraising and interpreting that evidence."*
Orme (2007) **[8]**	Public health for the 21st century : new perspectives on policy, participation, and practice *(Buch)*	Das Buch unterscheidet evidence-based und evidence-informed policy and practice im Kontext von Public Health, und definiert die Begriffe wie folgt:<br>• *“Evidence-based policy and practice refer to a situation where least judgement and risk is involved in the decision to use a specific intervention. The decision is based on clear evidence as to what the effectiveness of the intervention is likely to be; the evidence is most likely to be of an experimental nature. This is most likely to be relevant to practice decisions, or to policy decisions which are specific to a defined group or locality."*
Orme (2007) **[8]**		• *"Evidence-informed policy and practice refer to a situation where evidence has been used as a basis for a more complex judgement. The evidence may be of a wider nature, including, for example, evidence of social and economic conditions, and of the links between these and health"*

Gray et al. (2009) **[9]**	Evidence-based healthcare and public health *(Buch)*	*"The use of an evidence-based approach in public health practice and health promotion is the subject of much debate. Some practitioners hold the view that evidence-based is synonymous with using evidence from randomised controlled trials. Others are against the approach believing it to be inappropriate for public health because it is 'positivist' and 'decisionist'. However, to deny that public health decision should be based on evidence from research that is amplified, if possible, by evidence from experience is unrealistic. Furthermore, evidence about the costs and benefits of any public health or health promotion intervention must be related to the other needs of the population or community and the values that will also influence a decision (Fig. 8.6)."*
Gray et al. (2009) **[9]**		*"Knowledge-based public health will include not only evidence-based public health but also the traditional public health skills of data collection and analysis and the ability of public health practitioners to record and use the experience of communities and societies. Knowledge-based public health creates new opportunities for public health practice. (…) Just as public health services are responsible for monitoring and reporting on the quality and safety of water for their populations, they should also be responsible for monitoring, reporting on, and, if necessary, taking action to improve the quality and safety of the knowledge provided to their populations, including both professionals and members of the public."*
Satterfield et al. (2009) **[10]**	Toward a Transdisciplinary Model of Evidence-Based Practice *(Artikel)*	*"Our revised EBP model (…) has a transdisciplinary perspective. It incorporates each discipline's most important advances and attempts to address remaining deficiencies. The model is grounded in an ecological framework and emphasizes shared decision making. (…) The model's new external frame contains environment and organizational factors to create a cultural context that moderates the acceptability of an intervention, its feasibility, and the balance between fidelity and adaptation that is needed for effective implementation. Environment and organization are important to evidence-based decisions in all disciplines, although some disciplines, such as nursing, social work, and public health, may be more likely to choose or modify evidence-based interventions based on context. (…). With the goal of preventing disease in populations, public health interventions must be implemented through organizations and communities."*<br><br>*"Consistent with major EBM models, 'best available scientific evidence' remains one of the three circles. Evidence is defined as research findings derived from the systematic collection of data through observation and experimentation and the formulation of questions and testing of hypotheses."*

Brownson, Fielding, Maylahn<br><br>(2009)<br><br>**[11]**	Evidence-Based Public Health: A Fundamental Concept for Public Health Practice<br><br>*(Artikel)*	*"In summarizing these various attributes of EBPH, key characteristics include:*<br>- *Making decisions using the best available peer-reviewed evidence (both quantitative and qualitative research),*<br>- *Using data and information systems systematically,*<br>- *Applying program-planning frameworks (that often have a foundation in behavioral science theory),*<br>- *Engaging the community in assessment and decision making,*<br>- *Conducting sound evaluation, and*<br>- *Disseminating what is learned to key stakeholders and decision makers.*<br>*Accomplishing these activities in EBPH is likely to require a synthesis of scientific skills, enhanced communication, common sense, and political acumen."*
Gerhardus et al.<br><br>(2010)<br><br>**[12]**	Evidence-based Public Health<br><br>*(Buch)*	*"Evidence-based Public Health soll die Gesundheit auf Bevölkerungsebene durch wissenschaftlich abgesicherte Entscheidungen verbessern. Dafür wird das verfügbare Wissen der medizinischen, ökonomischen, ethischen, soziokulturellen und rechtlichen Aspekte von Krankheit und Maßnahmen systematisch, transparent und zielgerecht bewertet und in die Entscheidungsprozesse eingebracht. Alle Schritte - von der Problemstellung bis zur Umsetzung der Maßnahmen und Programmen - sollen explizit, transparent und begründet sein."*
European Centre for Disease Prevention and Control<br><br>(2011)<br><br>**[13]**	Evidence-based methodologies for public health<br><br>*(Bericht)*	*"Evidence-based public health could be defined as integrating the best available evidence with the knowledge and considered judgements from stakeholders and experts to improve health and protect the population from infectious and environmental hazards. "*

National Collaborating Centre for Methods and Tools (Canada)<br><br>(2011)<br><br>**[14]**	Evidence-Informed Public Health<br><br>*(Internetseite)*	*"What is evidence-informed public health? The process of distilling and disseminating the best available evidence from research, context and experience, and using that evidence to inform and improve public health practice and policy. Put simply, it means finding, using and sharing what works in public health.*<br><br>*Evidence-informed decision making in public health considers the valuable evidence from a variety of sources:*<br><br>• *community health issues and local context*<br>• *existing public health resources*<br>• *community and political climate*<br>• *the best available research findings*<br>*Decision makers must rely on their public health expertise to integrate all relevant factors into any conclusions or recommendations."*
Rychetnik et al.<br><br>(2012)<br><br>**[15]**	Translating research for evidence-based public health: key concepts and future directions<br><br>*(Artikel)*	*"Evidence-based policy and practice: policies and programmes that are explicitly informed by research evidence. This evidence may be derived from all stages of evidence-building and review, including effectiveness research, translation research on the replication and dissemination of programmes, as well as knowledge translation research on strategies to support the application of evidence."*
Vanagas, Bala, Lhachimi<br><br>(2017)<br><br>**[16]**	Evidence-Based Public Health 2017<br><br>*(Artikel)*	*"EBPH can be defined as a process of integrating evidence from scientific research and practice to improve the health of the target population. The key components of EBPH include making decisions on the basis of the best available scientific evidence and using sound data collection and research methods together with engaging the community in the decision-making. An EBPH approach could potentially have numerous direct and indirect benefits, including access to more and higher-quality information on best practice, a higher likelihood of successful prevention programs and policies, greater workforce productivity, and more efficient use of public and private resources."*

Brownson, Fielding, Green<br><br>(2018)<br>**[17]**	Building Capacity for Evidence-Based Public Health: Reconciling the Pulls of Practice and the Push of Research<br><br>*(Artikel)*	*“Evidence-based public health (or evidence-based decision making) [is] defined by several key characteristics that include making decisions based on evidence-based interventions; using data and information systems systematically; applying program planning frameworks; engaging the community in assessment and decision making; conducting sound evaluation; and disseminating what is learned to key stakeholders and decision makers.”*
Brownson, Fielding, Green<br><br>(2018)<br>**[17]**		*“Evidence-informed decision making [refers to] the process of distilling and disseminating the best available evidence from research, context, and experience (political, organizational) and using that evidence to inform and improve public health practice and policy."*
Center for Disease Prevention and Control<br><br>(n.a.)<br>**[18]**	Understanding Evidence<br><br>*(Internetdokument)*	*"Evidence Based Decision-Making is a process for making decisions about a program, practice, or policy that is grounded in the best available research evidence and informed by experiential evidence from the field and relevant contextual evidence."*

Evidenzbasierung in Gesundheitsförderung

Wiggers, Sanson-Fisher in Scott, Weston (Hrsg.)<br><br>(1998)<br><br>**[19]**	Evaluating health promotion<br><br>*(Buchkapitel)*	*„The planning and conduct of health promotion activities involves a staged decision-making process concerned with: the identification of health-related problems and needs in the community; the selection of interventions intended to redress these problems and needs; the assessment of intervention effectiveness and methods to achieve the widespread adoption of efficacious interventions. Evidence-based health promotion involves the explicit application of quality research evidence when making such decisions. Research evidence may be obtained from appraisal of the research literature or, where feasible, from research data collected by the health promotion practitioner. Evidence-based health promotion involves more than reading, appraising or collecting research data on an ad hoc basis, undertaken as a matter of interest of professional development. Rather, it involves the appraisal or collection of research data being systematically integrated into and directed by the health promotion decision-making process.“*

Smith, Tang, Nutbeam<br><br>(2006)<br><br>**[20]**	WHO Health Promotion Glossary: new terms<br><br>*(Artikel)*	*"The use of information derived from formal research and systematic investigation to identify causes and contributing factors to health needs and the most effective health promotion actions to address these in given contexts and populations."*<br>*"As a field which recognizes that health needs can be addressed by action at the individual, interpersonal, community, environmental and political levels, health promotion is informed by many types of evidence derived from a range of disciplines (Tang et al., 2003). These include epidemiological studies about health determinants, health promotion program evaluations, ethnographic studies about social and cultural influences upon health needs, sociological research about the patterns and causes of inequalities, political science and historical studies about the public policy making process and economic research about the cost-effectiveness of interventions. Among the applications of evidence to health promotion planning is the identification of health promotion outcomes and intermediate impacts that should be addressed in order to achieve the goals of health promotion actions (Nutbeam, 1998). It is important to note that formal evidence alone is not a sufficient basis for effective health promotion. External information can inform, but not replace the expertise of individual practitioners which guides the selection and application of evidence (Sackett et al., 1996; Tang et al.,2003)."*
Magnus, Knudtsen, Wist, Weiss, Lillefjell<br><br>(2016)<br><br>**[21]**	The search conference as a method in planning community health promotion actions<br><br>*(Artikel)*	*"An increased focus on evidence-based health promotion means that local communities should actively adopt evidence-based strategies in selecting and implementing health actions. This includes employing the best available scientific evidence, using data and information systematically, applying program-planning frameworks, engaging the community in decision-making, conducting sound evaluations, and disseminating the resulting knowledge (Brownson et al, 2009). Evidence-based strategies also involve the interplay of principles and values, ideology and evidence in decision-making. To accomplish evidence-based health promotion actions or programs, different types of approaches are necessary, such as leadership, community assessment, and participatory approaches (Brownson et al, 2009; Brownson et al, 1999). (...) Participation promotes a sense of belonging, engagement, and ownership within local communities, and a participatory approach is one of several elements of value found in the decision-making process for evidence-based health promotion. Participation has to do with the possible ways of receiving information or being involved in decision-making processes and/or self-determination."*

Tabelle Z2: Ausgewählte Charakteristika der Textauszüge

● Charakteristikum wird beschrieben
○ Charakteristikum wird nicht beschrieben

**AutorInnen**	**Bezeichnung**	**Bevölkerungs-orientierung**	**Beteiligte Disziplinen**	**Verständnis von Evidenz**			**Relevante Kriterien**	**Relevante Kompetenzen**	**Interessengruppen**
				Konzept	Aktualität	Qualität			
**Jenicek (1997) [1]**	evidence-based public health	Bevölkerungs-fokus	○	Breites Konzept	●	Beste Evidenz	○	○	ExpertInnen
**Brownson, Gurney, Land (1999) [2]**	evidence-based public health	○	Breites Spektrum	Breites Konzept	○	○	Mehrere Kriterien	●	ExpertInnen
**Canadian Health Services Research Foundation (2000) [3]**	evidence-based decision-making	○	○	Enges Konzept	○	Beste Evidenz	○	○	○

**Brownson**<br>**(2003)**<br>**[4]**	evidence-based public health	○	Breites Spektrum	Enges Konzept	○	○	Wirksamkeit	●	○
**Jenicek, Stachenko**<br>**(2003)**<br>**[5]**	evidence-based public health	Bevölkerungs-fokus	Breites Spektrum	Breites Konzept	●	Beste Evidenz	○	○	ExpertInnen inkl. Input der Zielpopulation
**Kohatsu, Robinson, Torner**<br>**(2004)**<br>**[6]**	evidence-based public health	○	○	Breites Konzept	○	○	○	○	ExpertInnen inkl. Input der Zielpopulation
**Rychetnik, Hawe, Waters, Barratt, Frommer**<br>**(2004)**<br>**[7]**	evidence based public health	○	Breites Spektrum	Breites Konzept	○	○	○	○	○
**Orme**<br>**(2007)**<br>**[8]**	evidence-based policy and practice	○	Enges Spektrum	Enges Konzept	○	○	Wirksamkeit	○	○
	evidence-informed policy and practice	Kontext-abhängigkeit	Breites Spektrum	Breites Konzept	○	○	○	○	○

**Gray (2009) [9]**	evidence-based approach in public health practice	Bevölkerungs-fokus	Breites Spektrum	Breites Konzept	○	○	Mehrere Kriterien	○	ExpertInnen inkl. Input der Zielpopulation
	knowledge-based public health	○	Breites Spektrum	○	○	○	○	●	ExpertInnen inkl. Input der Zielpopulation
**Satterfield et al. (2009) [10]**	evidence-based practice	Kontext-abhängigkeit	Breites Spektrum	Enges Konzept	○	Beste Evidenz	Mehrere Kriterien	○	Gemeinsame Entscheidungsfindung
**Brownson, Fielding, Maylahn (2009) [11]**	evidence-based public health	○	○	Breites Konzept	○	Beste Evidenz	○	●	Gemeinsame Entscheidungsfindung
**Gerhardus (2010) [12]**	evidence-based public health	○	Breites Spektrum	Breites Konzept	○	○	○	○	○
**European Centre for Disease Prevention and Control (2011) [13]**	evidence-based public health	○	○	Breites Konzept	○	Beste Evidenz	○	○	ExpertInnen inkl. anderer Entscheidungsträger

**National Collaborating Centre for Methods and Tools (Canada)** **(2011)** **[14]**	evidence-informed public health/ evidence-informed decision making in public health"	Kontext-abhängigkeit	Breites Spektrum	Breites Konzept	○	Beste Evidenz	○	●	ExpertInnen inkl. Input der Zielpopulation
**Rychetnik et al.** **(2012)** **[15]**	evidence-based policy and practice	○	Breites Spektrum	Enges Konzept	○	○	○	○	○
**Vanagas, Bala, Lhachimi** **(2017)** **[16]**	evidence-based public health	○	○	Breites Konzept	○	Beste Evidenz	○	○	Gemeinsame Entscheidungsfindung
**Brownson, Fielding, Green** **(2018)** **[17]**	evidence-based decision making	○	○	○	○	○	○	●	Gemeinsame Entscheidungsfindung
	evidence-informed decision making	Kontext-abhängigkeit	Breites Spektrum	Breites Konzept	○	Beste Evidenz	○	○	○

**Center for Disease Prevention and Control**<br><br>**(n.a.)**<br><br>**[18]**	evidence based decision making	Kontext-abhängigkeit	Breites Spektrum	Breites Konzept	○	Beste Evidenz	○	○	○
**Wiggers, Sanson-Fisher in Scott, Weston (Hrsg.)**<br><br>**(1998)**<br><br>**[19]**	evidence-based health promotion	Bevölkerungs-fokus	○	Enges Konzept	○	Gute Evidenz	Mehrere Kriterien	○	ExpertInnen
**Smith, Tang, Nutbeam**<br><br>**(2006)**<br><br>**[20]**	evidence-based health promotion	Mehrebenen-ansatz	Breites Spektrum	Breites Konzept	○	○	Mehrere Kriterien	○	○
**Magnus, Knudtsen, Wist, Weiss, Lillefjell**<br><br>**(2016)**<br><br>**[21]**	evidence-based health promotion	Bevölkerungs-fokus	○	Breites Konzept	○	Beste Evidenz	○	●	Gemeinsame Entscheidungsfindung

Literaturverzeichnis

1. Jenicek M (1997) Epidemiology, evidenced-based medicine, and evidence-based public health. J Epidemiol 7:187-197. 10.2188/jea.7.187
2. Brownson RC, Gurney JG, Land GH (1999) Evidence-based decision making in public health. J Public Health Manag Pract 5:86-97. 10.1097/00124784-199909000-00012
3. Canadian Health Services Research Foundation (2000) Health Services Research and Evidence-Based Decision-Making. In: https://www.cfhi-fcass.ca/migrated/pdf/mythbusters/EBDM_e.pdf. 04.07.2019
4. Brownson RC (2003) Evidence-based public health. Oxford University Press, New York; Oxford
5. Jenicek M, Stachenko S (2003) Evidence-based public health, community medicine, preventive care. Medical science monitor : international medical journal of experimental and clinical research 9:SR1-7. PMID 12601308
6. Kohatsu ND, Robinson JG, Torner JC (2004) Evidence-based public health: an evolving concept. Am J Prev Med 27:417–421. 10.1016/j.amepre.2004.07.019
7. Rychetnik L, Hawe P, Waters E, Barratt A, Frommer M (2004) A glossary for evidence based public health. J Epidemiol Community Health 58:538–545. 10.1136/jech.2003.011585
8. Orme J (2007) Public health for the 21st century: New perspectives on policy, participation, and practice. Open University Press, Maidenhead, Berkshire, England; New York
9. Gray JaM, Shepperd S, Ison E, Lees R, Pearce-Smith N (2009) Evidence-based healthcare and public health: How to make decisions about health services and public health. Churchill Livingstone Elsevier, Edinburgh
10. Satterfield JM, Spring B, Brownson RC et al. (2009) Toward a Transdisciplinary Model of Evidence-Based Practice. The Milbank Quarterly 87:368–390. 10.1111/j.1468-0009.2009.00561.x
11. Brownson RC, Fielding JE, Maylahn CM (2009) Evidence-based public health: a fundamental concept for public health practice. Annu Rev Public Health 30:175-201. 10.1146/annurev.publhealth.031308.100134
12. Gerhardus A, Breckenkamp J, Razum O, Schmacke N (2010) Evidence-based Public Health. Hans Huber, Bern
13. European Centre for Disease Prevention and Control (2011) Evidence-based methodologies for public health – How to assess the best available evidence when time is limited and there is lack of sound evidence. In:European Centre for Disease Prevention and Control, Stockholm https://www.ecdc.europa.eu/sites/portal/files/media/en/publications/Publications/1109_TER_evidence_based_methods_for_public_health.pdf. 19.07.2019
14. National Collaborating Centre for Methods Tools (2011) Evidence-Informed Public Health. 17.11.2020, https://www.nccmt.ca/about/eiphhttps://www.nccmt.ca/about/eiph
15. Rychetnik L, Bauman A, Laws R et al. (2012) Translating research for evidence-based public health: key concepts and future directions. J Epidemiol Community Health 66:1187–1192. 10.1136/jech-2011-200038
16. Vanagas G, Bala M, Lhachimi SK (2017) Evidence-Based Public Health 2017. Biomed Res Int 2017:2607397. 10.1155/2017/2607397
17. Brownson RC, Fielding, J. E., Green, L. W. (2018) Building Capacity for Evidence-Based Public Health: Reconciling the Pulls of Practice and the Push of Research. Annu Rev Public Health 39:27–53. 10.1146/annurev-publhealth-040617-014746
18. Center for Disease Control and Prevention Understanding Evidence: Evidence Based Decision Making Summary. 17.11.2020, https://vetoviolence.cdc.gov/apps/evidence/docs/EBDM_82412.pdfhttps://vetoviolence.cdc.gov/apps/evidence/docs/EBDM_82412.pdf
19. Wiggers J S-FR (1998) Evidence-based health promotion. In: Scott D, Weston R (eds) Evaluating health promotion. Nelson Thornes, Cheltenham, U.K., p 126–145

20. Smith BJ, Tang KC, Nutbeam D (2006) WHO Health Promotion Glossary: new terms. Health Promot Int 21:340–345. 10.1093/heapro/dal033
21. Magnus E, Knudtsen MS, Wist G, Weiss D, Lillefjell M (2016) The Search Conference as a Method in Planning Community Health Promotion Actions. Journal of public health research 510.4081/jphr.2016.621
